# One Size Does Not Fit All: Heterogeneity in Developmental Hematopoiesis

**DOI:** 10.3390/cells11061061

**Published:** 2022-03-21

**Authors:** Cristiana Barone, Roberto Orsenigo, Raffaella Meneveri, Silvia Brunelli, Emanuele Azzoni

**Affiliations:** School of Medicine and Surgery, University of Milano-Bicocca, 20900 Monza, Italy; cristiana.barone@unimib.it (C.B.); r.orsenigo@campus.unimib.it (R.O.); raffaella.meneveri@unimib.it (R.M.); silvia.brunelli@unimib.it (S.B.)

**Keywords:** hematopoiesis, heterogeneity, embryo, hemogenic endothelium, HSC, EMP, LMP

## Abstract

Our knowledge of the complexity of the developing hematopoietic system has dramatically expanded over the course of the last few decades. We now know that, while hematopoietic stem cells (HSCs) firmly reside at the top of the adult hematopoietic hierarchy, multiple HSC-independent progenitor populations play variegated and fundamental roles during fetal life, which reflect on adult physiology and can lead to disease if subject to perturbations. The importance of obtaining a high-resolution picture of the mechanisms by which the developing embryo establishes a functional hematopoietic system is demonstrated by many recent indications showing that ontogeny is a primary determinant of function of multiple critical cell types. This review will specifically focus on exploring the diversity of hematopoietic stem and progenitor cells unique to embryonic and fetal life. We will initially examine the evidence demonstrating heterogeneity within the hemogenic endothelium, precursor to all definitive hematopoietic cells. Next, we will summarize the dynamics and characteristics of the so-called “hematopoietic waves” taking place during vertebrate development. For each of these waves, we will define the cellular identities of their components, the extent and relevance of their respective contributions as well as potential drivers of heterogeneity.

## 1. Introduction

The notion that the adult hematopoietic system is inherently heterogeneous has been appreciated for a long time. More than 10 blood cell types with diverse functions, ranging from innate and acquired immunity (leukocytes) to O_2_ and CO_2_ transport (erythrocytes) and hemostasis (megakaryocytes, platelets) are generated from hematopoietic stem cells (HSCs), normally residing in the bone marrow (BM). Although less well studied than the adult, hematopoiesis in the developing embryo is characterized by several additional layers of heterogeneity, represented by the generation of hematopoietic cells in multiple overlapping waves, originating at very specific times from both extraembryonic and intraembryonic sites, and by the presence of stem and progenitor cells unique to fetal life. The functional meaning of this diversity has been an active area of research in the last decades, and is still incompletely understood. The recent technological advances in single-cell multi-omics and imaging have allowed investigators to explore the heterogeneity of the developing hematopoietic system at a resolution that was practically impossible to obtain until few years ago. To date, the resulting picture is a very complex and dynamic process that has important implications for adult physiology and disease, which would not have been predicted until recently. This review will focus on untangling the different aspects of cellular heterogeneity during embryonic hematopoiesis, with a special eye on interpreting the diversity in stem and progenitor cell populations.

## 2. The Origins of Hematopoiesis: Hemangioblast and Hemogenic Endothelium

### 2.1. Before the Hemogenic Endothelium: The Hemangioblast Theory

The existence of progenitor cells capable of differentiating into both endothelial and hematopoietic cells (“hemangioblasts”) was initially suggested by Florence Sabin in 1917, based on microscopical observations which noted the close physical proximity of emerging endothelial and red blood cells in the yolk sac (YS) of chicken embryos. The actual “hemangioblast” denomination was coined by Murray in 1932 [1] and referred to a mass of cells derived from the primitive streak mesoderm, containing precursors of endothelium and blood cells. The first experimental data that suggested the presence of a hemangioblast did not arrive until the 1990s when Gordon Keller and colleagues identified a clonal mesodermal precursor for blood and endothelium in embryonic stem (ES) cell differentiation cultures, the blast colony-forming cell (BL-CFC) [2]. This work introduced a paradigm shift in which the hemangioblast no longer represented a population of cells as initially implied by Murray, but a bipotent clonal progenitor for blood and endothelium. The same laboratory went on to provide in vivo evidence of the presence of hemangioblasts in mouse embryos, first detected at the mid-streak stage of gastrulation (E6.75) [3]. Labeling of cells fated to endothelial and hematopoietic lineages in zebrafish was also interpreted as in vivo support for the existence of the hemangioblast [4]. However, in the same years, several experimental evidences started lending support to another theory for the origin of blood cells in the embryo: the hemogenic endothelium (discussed in the following section), which partially clashed with the hemangioblast [5]. After few years, a study based again on ES cultures tried reconciling the two apparently conflicting hypotheses by providing a model in which the hemangioblast gives rise to blood cells through a hemogenic endothelial intermediate [6]. More recent work using lineage tracing of individual cells in the mouse epiblast, primitive streak, and early YS provided little support for the in vivo existence of hemangioblasts as the majority of labeled clones contained either blood or endothelial cells, but very few clones harbored both lineages [7]. These results suggest that precursors for primitive blood and YS endothelium are already specified before gastrulation. Comparison of these lineage tracing data with the BL-CFC model, which was supported primarily by ES cultures, serves as a reminder that in vitro potential does not always equal in vivo fate, which is often more restricted. The hemangioblast may therefore represent the in vitro counterpart of an early mesodermal precursor cell present prior to the commitment to the hematopoietic lineage, as opposed to an actual bipotent progenitor persisting in vivo until midgestation. Further investigation and new experimental approaches are needed to clarify this issue.

### 2.2. Heterogeneity of the Hemogenic Endothelium

Several landmark studies in the 1990s provided strong evidence that in vertebrates definitive repopulating HSCs are autonomously generated intra-embryonically within the developing Aorta-Gonad-Mesonephros region (AGM) at midgestation, prior to their appearance in other hematopoietic organs [8,9,10,11,12,13,14]. HSCs and their precursors (pro- and pre-HSCs) are initially found within clusters of hematopoietic cells observed in the aorta and major embryonic arteries [15,16,17,18,19]. Similar to the putative hemangioblasts, these peculiar cell clusters were first identified microscopically in the beginning of the last century, and their close association to the vascular endothelium lent support to the idea that the embryonic endothelium has a hemogenic capacity (reviewed in [20]). Later lineage tracing studies in the chick and mouse models confirmed that this was indeed the case [21,22]. The strongest support for an endothelial origin of hematopoietic cells came from more recent live imaging studies that visualized the transition of endothelium into blood in real-time, both in vitro [23] and in/ex vivo [24,25,26]. The process of endothelial cells acquiring a hematopoietic fate has been termed endothelial-to-hematopoietic transition (EHT). Cells undergoing this process experience major morphological changes independent of cell division, including breakage of tight junctions with adjacent endothelial cells, which result in their acquisition of a round shape and the expression of a hematopoietic program. Importantly, hemogenic endothelial cells are not bipotent but rather already committed to an hematopoietic fate [27,28]. How EHT is regulated at the molecular level is complex and still incompletely understood, though the requirement for the master hematopoietic transcription factor Runx1 is well established (reviewed in [29]). Although EHT has been mostly studied in the context of the dorsal aorta because of it being the primary site of HSC generation, it is now clear that a similar process also takes place in other locations, such as YS, placenta, and possibly other sites [30,31]. Hemogenic endothelium (HE) is inherently transient and it is present only during a limited timeframe in embryonic development, although a potential wave with limited hematopoietic contribution has been described also in the BM of late fetus/young adults [32]. HE represents a common source of various types of stem and progenitor cells generated in multiple anatomical locations at different times during embryogenesis, including progenitors with broad mesodermal potential [33]. This poses an important question: are prospective hematopoietic cells intrinsically fated to become a specific type of stem or progenitor cell already at the HE stage, or do they become specified only later, in response to extrinsic cues from the microenvironment? In other words, when exactly is heterogeneity generated during developmental hematopoiesis? This is a basic research question which carries important translational relevance. Achieving a better understanding of the embryonic hematopoietic system roadmap will inform experimental strategies for production of hematopoietic stem and progenitor cells (HSPCs) ex vivo, which have recently been reported, though their efficiency is still relatively low [34,35].

There is evidence in support of the notion that HE is intrinsically heterogeneous. Clonal assays of individual hemogenic endothelial cells demonstrated very high heterogeneity in their ex vivo output at a given developmental stage [36]. HE could therefore consist of a mix of precursors endowed with distinct potential, and/or that differentiation process proceeds in a highly asynchronous way. These two hypotheses do not exclude each other–and, in fact, there is experimental evidence supporting both. As mentioned above, the formation of HSCs requires the transcription factor Runx1, but also its non-DNA binding partner core binding factor β (CBFβ). The same requirement is observed for the generation of Erythro-Myeloid Progenitors (EMPs), multipotent progenitors distinct and appearing earlier than HSCs. A first demonstration that EMPs and HSCs differentiate from distinct populations of hemogenic endothelial cells came from a study using a CBFβ rescue strategy, which showed that when CBFβ was reintroduced in a null background under the control of two different tissue specific expression transgenes, this caused the alternate rescue of EMPs or HSCs, but not of both [37]. A recent study supported and extended these findings [38]. The authors employed single cell index sorting of HE combined with a co-culture strategy to show that rare HSC-competent HE and the relatively more abundant progenitor-restricted HE can be discriminated by expression of the chemokine receptor CXCRSingle cell RNA-Seq (scRNA-Seq) of the two HE types isolated from E9–E9.5 mouse embryos showed that the HSC competent HE already expresses many of the genes known to be enriched in E11.5 AGM HSCs [38,39]. Accordingly, CXCR4 lineage tracing has been recently used to mark a contribution of intra-but not extra-embryonic HE to innate lymphoid cells [40] and HSC-derived monocytes from microglia and other tissue-resident macrophages (TRM) [41]. Recently, some markers enriched in the YS HE have also been identified, such as Stab2 and Lyve1 [42,43], although the latter was shown not to be exclusively expressed in the YS [44]. These data support the concept that HE cells are already “primed” to become specific types of stem or progenitor cells when they are still undergoing EHT. What are the signals that instruct the generation of heterogeneity in prospective hemogenic endothelial cells?

It is now clear that different signaling pathways are involved in EMP and HSC formation (Table 1). For example, EMP formation from HE in the YS vascular plexus does not require Notch signaling, whereas HSC production is strictly dependent on Notch [45,46,47]. The Notch pathway plays a fundamental role in the arterialization of HE, and the arterial identity of HE is a mandatory condition for the establishment of HSC generation [48,49], but not for EMPs in the YS, as they emerge from both arterial and venous endothelium [31]. The arterial identity of HE appears to be important not only for HSC emergence, but more in general for the appearance of progenitors with lymphoid potential [50]. If the dependence on Notch pathway can be considered a discriminant between HSC-fated HE in the embryo proper and the HE generating EMPs in the YS, the WNT canonical pathway is instead a common regulator of both programs [31]. Other signaling pathways appear to be differentially required in distinct types of HE. In particular, the emergence of HSCs requires mechanistic stimuli provided by the onset of circulation and blood flow, which result in the modulation of several important pathways, among which nitric oxide (NO) [51], cAMP and BMP [52,53], Rho-Yap [54] and metabolic pathways [55]. In contrast, EMP emergence is blood flow-independent [56]. Interestingly, ex vivo modulation of hypoxia/glycolysis yielded opposite effects in embryo proper and YS cells, with a decrease of the hematopoietic output in the former and an increase in the latter [55]. Hepatic leukemia factor (Hlf) is another discriminating factor between EMPs and HSCs [57]. As determined by analysis of a transgenic reporter mouse model, Hlf is expressed by virtually all intra-embryonic c-Kit^+^ cells, including HE and hematopoietic clusters in the dorsal aorta and vitelline artery, but not by non-hemogenic endothelium and E9.5 YS EMPs. Intriguingly, Hlf appears to be also expressed by some c-Kit clusters in the E10.5 YS. These results are another strong suggestion that the acquisition of the HSC or progenitor fate takes place at an early stage during hematopoietic differentiation.

Many studies have very recently employed scRNA-Seq and newer variants of this technique such as spatial transcriptomics to delve deeper into the study of the molecular determinants of EHT and to gain insight into the cellular and molecular heterogeneity of HE [39,58,59,60,61,62,63,64], mostly in animal models but some also in human [65,66]. These studies mainly focused on intra-embryonic EHT because of its obvious connection with HSC generation, and collectively highlighted how (pro-/pre-)HSC development proceeds in a fairly asynchronous way, with cells at different stages of maturation coexisting in the same embryo. scRNA-seq allowed to describe the differentiation trajectory of EHT in greater detail by revealing previously unrecognized intermediate stages, such as the “pre-HE”, which may represent a developmental bottleneck in which many important molecular pathways are active [58]. Moreover, these studies confirmed the heterogeneity within the intra-aortic clusters population by highlighting the presence of lympho-myeloid biased progenitors, which can be distinguished molecularly [58]; however, the precise cellular relationships between these different progenitor types and the HE are still unclear. Single cell studies not only identified unexpected differences, but also similarities. Indeed, non-HE and HE show surprisingly similar transcriptomes with only a few differentially expressed genes (DEG) [59]; this was confirmed by another study showing that HE cells clustered mainly according to the tissue of origin and to a lesser extent according to hemogenic/non hemogenic identity [67]. In a similar way, clusters in the ventral and dorsal aspects of the aorta also exhibited few global transcriptional differences [59]. Transcriptomics studies have recently been instrumental in identifying microenvironmental cues as driving forces of heterogeneity in the embryonic hematopoietic niche [62,65,68]. These and other studies [69,70,71] showed that secreted factors (BMP, Kit ligand, Noggin as ventral signals; Shh as a dorsal signal are among the most well-characterized) contribute to generating the dorso/ventral polarization of signals that places the embryonic (pre-)HSC niche in the ventral aspect of the aorta.

## 3. Heterogeneity of Hematopoietic Stem Cell (HSC)-Independent Hematopoiesis

### 3.1. Multiple Waves of HSC-Independent Progenitors

The role of HSCs during embryonic and fetal development has been recently subject of a paradigm shift. Recent studies analyzed the extent of their contribution to pre-natal hematopoiesis and, surprisingly, concluded that they are less important than previously thought. Indeed, seminal work revealed that so-called HSC-independent embryonic hematopoietic progenitors are necessary and sufficient to sustain embryonic hematopoiesis up until birth [37,72]. In the past, the primary role of HSC-independent hematopoiesis was thought of consisting in the supply of oxygen for the developing embryo, a task performed by primitive erythrocytes. Now, it has been shown that HSC-independent myelopoiesis and lymphopoiesis also carry on several vital functions. TRM generated as part of these lineages can support the organization of vascular networks, direct fetal organogenesis and even promote HSC formation [73]. Moreover, despite HSC-independent progenitors having long been considered transient, it has emerged that their progeny can in fact persist until adulthood and self-renew independently of HSCs [73].

As mentioned above, embryonic hematopoietic cells arise in consecutive and overlapping waves. In mice, the first wave arises extra-embryonically between the mid-streak (E7.0) and the neural plate stage (E7.5) and is called “primitive”, to distinguish it from the subsequent two waves often termed “definitive” [74], a definition initially given based on distinct globin expression patterns. Beyond erythrocytes, also megakaryocytes (Mk) and macrophages (Mac) emerge concomitantly in the YS and therefore have been considered part of the same wave [75]. However, the cellular origins of the primitive wave are overall still unclear. Whereas the appearance of primitive erythrocytes likely precede HE specification, a notion reinforced by the observation that primitive erythrocytes are present in Runx1^-/-^ embryos in which EHT is impaired [76], the precise origin of primitive Mk and Mac has not yet been clearly defined [77]. The second wave of HSC-independent progenitors (which can be addressed as “pro-definitive”) originates from HE in the YS starting from around E8.25, concomitantly with the onset of circulation, and consists in the emergence of multi-potent progenitors among which are EMPs [74,78] and lympho–myeloid progenitors (LMPs), the latter appearing slightly later (around E9.5) [79]. This timeline also coincides with the appearance of HSC-independent T- and B- innate-type lymphoid progenitors, which display a heterogeneous origin as they can derive from both intra- and extra-embryonic HE [80,81]. The lineage relationship of these progenitors with LMPs awaits better clarification [82]. The generation of HSC precursors from HE in the embryo proper marks the onset of the third and definitive hematopoietic wave. It is generally accepted that this wave starts in the AGM region at E10.5 because this is the first time and location in development in which transplantable HSCs are detected [8], although pro-HSCs can be found in the para-aortic splanchnopleura (P-Sp) already at E9.5 [16]. Most pre-HSCs do not complete their differentiation in the AGM, but instead colonize the liver, where they undergo maturation into fully functional transplantable HSCs [83].

Judging from the complex picture briefly summarized above, it is easy to understand that the current state of knowledge of the developing hematopoietic system makes talking about the different waves using the “primitive” and “definitive” terminology far too simplistic. These two terms may be in fact better suited to indicate unipotent and multipotent progenitors, as previously suggested [77]. As mentioned, at least three major hematopoietic waves are recognized in the mammalian embryo. However, the reality is probably even more complex, as the cells belonging to each of these three major waves are in fact very heterogeneous and consist of cells with different properties and in vivo fates, despite having a similar origin (timing/location), and in some cases a similar surface marker phenotype. These last two facts, together with the added difficulty that the three waves mix in space and time due to the onset of circulation, have hampered the understanding of the real heterogeneity of the developing hematopoietic system, which is only now starting to surface in its true complexity, thanks to new technological advances. In the next paragraphs, we will try to summarize the current state of knowledge in the field.

### 3.2. “Primitive” Hematopoiesis: One Wave, Different Origins?

**Figure 1:**
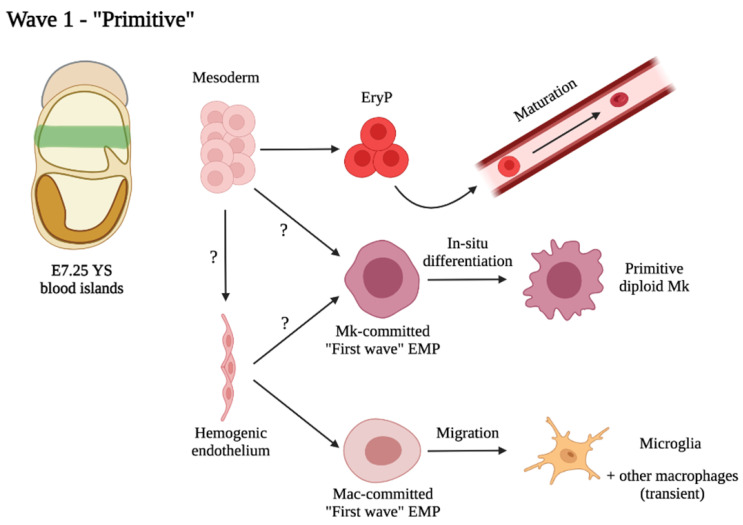
Proposed schematic depicting the primitive wave of hematopoiesis.

The hematopoietic cell lineages grouping under the definition of “primitive” comprise erythrocytes, Mk and Mac (Figure 1). Large nucleated erythroid cells were detected in the YS blood islands during pioneering studies of already more than a century ago (reviewed in [84]). Primitive erythrocytes (EryP) have unique cellular and molecular features that easily enable to distinguish them from definitive erythrocytes (EryD). They are nucleated and much larger than definitive ones [84]. Furthermore, primitive erythrocytes express embryonic form of globins (εy- and βH1- globins in the mouse) [85]. Later studies of carefully staged mouse embryos revealed the presence of unique erythroid progenitors termed EryP-CFC emerging in the YS at E7.25, peaking in numbers at E8.25 and shortly afterwards becoming no longer detectable [74]. Similar to definitive erythroid cells, primitive erythroid cells ultimately undergo terminal differentiation between E12.5 and E16.5 in the mouse, which involves progressive reduction in cell size, enucleation, accumulation of hemoglobin and loss of mitochondria [86]. This process takes place directly in circulation, as opposed to definitive erythroid precursor of the subsequent wave, which mature in the fetal liver (FL). Differentiated primitive erythroid cells are still detectable for a few days after birth [86]. The cellular origin of primitive erythrocytes is still debated. As mentioned above, the most accredited theory so far has been that primitive erythroid cells emerge directly from mesoderm soon after gastrulation. However, recent studies mainly using ES cell cultures as a model have questioned this notion by suggesting that primitive erythrocytes may be generated through a HE(-like) intermediate [87,88]. These data apparently clash with the fact that primitive erythrocytes are still generated in normal numbers in the absence of Runx1, master regulator of EHT [89]. If this is confirmed, it would mean that this putative early HE committed to the primitive erythroid lineage functions through a yet unidentified Runx1-independent mechanism, and that the HE is more heterogenous than currently thought. However, Runx1 is not entirely dispensable for this lineage, as in its absence primitive erythrocytes are morphologically and functionally defective [90].

The first Mk progenitors can be detected in the YS at E7.The close overlap in space and time of the Mk lineage with primitive erythroid cells led to the hypothesis that these two lineages shared a common progenitor. Indeed, megakaryocyte-erythroid bipotential progenitors (MEPs) were identified already in mid-primitive streak embryos, along with EryP-CFC and Mk-restricted progenitors [91] and are distinct from later MEPs which seed the FL and associate with the definitive erythroid wave. The Mk lineage displays heterogeneity from its onset: the first cells able to produce platelets, identified by high expression of CD41 and GP1bb, are diploid, smaller in size than later Mk (which are bigger and polyploid), and appear to be generated, at least in part, via a Runx1-, progenitor- independent pathway [92]. Hence, these cells may represent the true “primitive” Mk, in contrast with later MEP-dependent ones, and similar to primitive erythrocytes, they were thought to derive from hemangioblast-like precursors [91]. Interestingly, a recent study, employing a pulse-chase lineage tracing approach combined with scRNA-Seq, identified a Myb-independent pathway in which EMPs (typically associated with the second hematopoietic wave) generate Mk in the YS through direct differentiation bypassing the MEP stage [93]. It is unclear how these Myb-independent Mk relate to the previously identified diploid primitive Mk [92], but, based on timing and location of appearance and the surface marker phenotype, we speculate that they may represent the same cells. If this is the case, this would imply that EMPs are not exclusively involved in the definitive-type hematopoiesis, but also take part in the primitive wave, thus prompting a revisitation of the association of the various progenitors to the different waves.

Mac progenitors are first detected in the YS starting from E7.25 in the mouse [74], in a spatial and temporal fashion overlapping with that of the primitive erythroid cells and Mk. The very first Mac, however, are derived from the mother [94]. Shortly afterwards, monopotent Mac precursors appear in the YS and differentiate from c-Kit^+^ progenitors bypassing monocyte intermediates [94]. Mac are completely absent in Runx1^-/-^ ES cell cultures and embryos [89,95], so it would appear that all Mac generated in the embryo originate through EHT. The first Mac wave arises independently of c-Myb [96], similar to the progenitors generating the first Mk. What is the progenitor of this primitive wave of embryonic Mac? As c-Kit in the YS is a marker of EMPs [78], there could be a first wave of “early” EMPs that are fated to differentiate to Mac [97]. The role of these primitive Mac was long believed to consist essentially in tissue remodeling and phagocytosis of dead cells. As early as E9, primitive Mac colonize all tissues, starting from the brain [98]. They comprise the predominant Mac population in most organs until E13.5, but are later replaced by monocyte-derived Mac, with one notable exception: brain microglia [97]. Although it is now accepted that microglia in mammals remain of extraembryonic origin throughout life with little/no contribution from monocyte-derived Mac [99], their precise origin has been subject of debate. Do microglia originate from primitive Mac [100,101] or from early EMPs [97,102,103]? Is it possible that we are calling the same cells with different names? Progenitors of microglia have a CD45^−^ c-Kit^+^ immunophenotype, the same as the progenitors of primitive Mac [94,102]. Pulse-chase fate-mapping of HE at E7.5 results in labelling of progenitors fated to microglia, as well as progenitors of TRMs, known to originate from *bona fide* EMPs [103,104]. However, we demonstrated that microglia progenitors are generated independently from the c-Kit/SCF pathway, whereas all other TRMs require this pathway for their normal development, lending support to the hypothesis that the progenitors of microglia are different from the EMPs that produce other TRMs [70]. One hypothesis that would reconcile these apparently contradicting observations is that microglia (at least initially) originate from a first wave of Mac-committed progenitors that are generated through EHT and locally undergo direct differentiation into Mac in the YS (thus not requiring c-Kit for expansion in the FL); after that, they quickly go on to colonize the developing brain where they acquire microglia characteristics. The primitive Mac/EMP confusion may have originated from the fact that most studies analyzing the ontogeny of embryonic Mac relied on fate mapping approaches that labelled both primitive progenitors and EMPs, due to lack of specificity of promoters used (Csf1r, Runx1, Cdh5 are expressed by both EMPs and primitive progenitors) and the fact that the two waves overlap in space and time [77]. The “EMP” definition therefore may have been incorrectly extended to microglia progenitors– indeed, the erythro/myeloid potential of these cells was not assessed at single cell level [102], but the data supporting the hypothesis are also consistent with these cells only displaying Mac potential in vivo [100]. The same can be said for the EMPs originating Mk [93]: is it correct to call these cells “erythro-myeloid progenitors” when they only display Mk potential in vivo? Nevertheless, what EMPs and primitive Mac may have in common, together with the progenitors of primitive Mk, is that they all appear to be generated from a HE—that may be inherently heterogeneous, as discussed in the previous paragraph. For these reasons, we still favor the “primitive” nomenclature to designate monopotent progenitors of each of the three lineages [77], though it has to be kept in mind that this definition may be used to define progenitors with different origins (mesoderm for erythroid cells and HE for Mk and Mac).

### 3.3. The “Second Wave”: Appearance of Multi-Potent Hematopoietic Progenitors in the Embryo

For more than 20 years, it has been recognized that multi-potent hematopoietic progenitors appear in the YS region starting at E8.25, following the primitive wave [105] and that this site harbors two erythroid waves synthesizing embryonic and adult-type globins [106]. The first detailed study mapping a precise timeline of progenitor generation in the YS came from J. Palis and colleagues, who demonstrated by means of colony-forming assays that progenitors bearing definitive-type erythroid and myeloid potential were generated with a different kinetic than that of primitive hematopoietic cells [74]. These second wave progenitors were then termed “HPP-CFC” because of their high proliferative potential and some extent of self-renewal [107]. Interestingly, mast cell progenitors were arising in a similar fashion in colony forming assays [74]. At the time, the lineage relationship of these progenitors with HSCs was unclear because their surface marker phenotype (CD45^low/-^ c-Kit^+^ AA4.1^+^) was very similar to the one of HSCs purified from the embryo proper [94,108] and lymphoid potential, initially considered a hallmark of HSC features, was also detected within these cells [105]. The precise identity of the second wave multipotent YS progenitor subset, and in particular the argument whether these cells were HSCs or HSC-derived, or instead represented committed progenitors spawned a long debate over the last decades, with prevailing evidence now supporting their HSC-independent nature [109]. Indeed, it is now clear that the second YS wave does not comprise HSCs or HSC precursors [16,44], although similar to HSCs, these cells arise from HE (see above). At the same time, new evidences recently allowed the true relevance of this wave of progenitors to start surfacing, as it is now accepted that they are necessary and sufficient for embryonic survival at least until birth [37,72]

**Figure 2:**
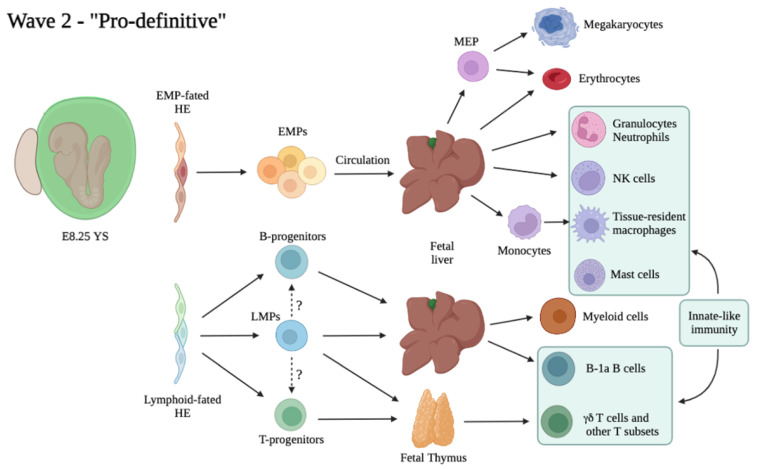
Proposed model of the second, or “Pro-definitive” hematopoietic wave. Dotted lines and “?” symbol indicate unclear lineage relationships between progenitors.

What was previously known as “multipotent progenitor” or “HPP-CFC” is now often addressed as EMPs (Erythro-Myeloid Progenitors), as these cells are a major component of the second, or “pro-definitive” hematopoietic wave (Figure 2). Further studies from the Palis group characterized the EMP surface marker phenotype as c-Kit^+^ CD31^+^ CD41^+^ CD16/32^+^ Sca1^−^, and showed that this subset comprises all CFU potential within the E9.5 YS [78]. The authors assayed EMP potential at the single cell level and showed that they comprise a very heterogeneous population with, at least ex vivo, different potential: erythroid, myeloid (granulocyte or Mac), and Mk, in various combinations. Similar to HE, this variegated output could be a result of intrinsic heterogeneity, different stages of maturation or a bias generated in culture. Importantly, EMPs lacked B-lymphoid potential overall, and they could only provide short-term erythroid reconstitution when transplanted into wild type or immunocompromised recipients [78]. After their initial generation in the YS, EMPs seed the FL where they expand and sustain fetal erythro-myelopoiesis up until birth [70,72,103]. Recently, EMPs were identified as the source of TRM, which are long-lived and can self-renew in the adult independent of HSCs [97,103,110,111]. TRMs are found within most tissues in relatively small numbers, and together with a common activity of performing phagocytosis and roles in tissue surveillance and homeostasis, have evolved specialized functions dependent on the tissue of residence, such as recycling erythrocytes in the liver (Kupffer cells) or spleen (red pulp Mac), repurposing surfactant in the lung (alveolar Mac) and remodeling synapses (brain microglia) [112]. Another example of heavily adapted Mac are osteoclasts, giant multinucleated cells of the Mac lineage, involved in bone resorption and remodeling. Two recent studies showed that they are also derived from EMPs [113,114]. It is surprising to think that despite the high level of heterogeneity in morphology and functions of TRM, they all arise from a common progenitor, the EMP. A central question in the field has been to determine how this heterogeneity is generated. A recent study employed scRNA-Seq to provide such insight, and demonstrated that a core Mac program is initiated early in EMPs, including chemokine, cytokine, pattern recognition, Fc gamma and phagocytic receptors, which results in the differentiation of “pre-macrophages” (pMacs) already at E9.5 in the mouse. These cells then distribute through the embryo during organogenesis and simultaneously complete their differentiation via a process determined by tissue-specific transcriptional regulators, thus generating adult TRM diversity [115]. As EMPs were shown to generate monocyte-derived TRM after seeding the FL [97], it is not clear how this process differs between Mac originating by unipotent progenitors as part of the first wave and Mac produced after FL colonization. The pool of adult Mac can be replenished from HSC-derived monocytes at variable rates in different tissues. However, specific functions of EMP-derived TRMs can be different than those of HSC-derived Mac. Indeed, accumulating evidence shows that the acquisition of some functions in Mac precursors can be specifically dictated by ontogeny. For example, although HSC-derived monocytes can produce cells resembling microglia in morphology and surface markers, only YS derived cells can generate true, fully functional microglial cells [116]. Another example concerns specification of Kupffer cells in the liver, in which the critical ability of performing uptake and recycling of erythrocytes can’t be acquired when these cells differentiate from monocytes [117]. Importantly, TRM development appears to be highly conserved between human and mouse, lending support to the findings obtained using animal models [118]. In addition to Mac, it has been reported that at least some other components of the innate immune system, natural killer (NK) cells, are initially derived from EMPs. Similar to Mac, EMP-derived NK cells exhibit ontogeny-specific characteristics, and in particular they are larger and more prone to cytotoxic degranulation response in comparison to their adult counterparts [119]. Genetic or environmental perturbations of EMPs can result in adult disease, such as histiocytosis or neurodegeneration, as recently modeled in mice [120].

Given their role as the progenitors of multiple hematopoietic populations critical for embryonic and adult survival, it is interesting to determine whether EMPs are heterogeneous in origin or whether their heterogeneity is generated through a mechanism similar to the one that generates TRMs diversity in EMP-derived pMacs, i.e., through environment-derived signals. Nevertheless, to date there is little information about EMP heterogeneity. To this aim, a recent study has employed a scRNA-Seq approach in combination with Csfr1-based lineage tracing of E9.5/E10.5 YS labelled cells [93] Gene expression analysis identified myeloid-primed and erythroid-primed EMPs, as well as Mk-biased EMPs specific to the E9.5 stage which likely represent progenitors of primitive Mks discussed in the previous paragraph [93]. These data demonstrate that as early as E10.5 a large fraction of EMPs has initiated their commitment to a specific lineage, but it is unclear what are the signals that generate the lineage bias already seen in YS EMPs. Although EMPs are known to respond to cytokines also found in adult hematopoiesis [121], very few YS niche signals have so far been identified; one such factor is Kit ligand, which triggers EMP expansion already in the YS [70]. It will be interesting to investigate what other extrinsic factors are specifically present in the YS microenvironment, and may play a role in determining EMP fate choice.

B- and T-lymphoid cells were initially thought to represent a hallmark of HSC-derived hematopoiesis. However, precursors of B and T lymphoid cells have been identified in the E8.5 and E9.5 mouse YS since the early 90s [122,123]. Using a co-culture approach, it was later demonstrated that progenitors isolated from the YS and the P-Sp prior to HSC emergence or in embryos devoid of HSCs could produce B-1a, but not B-2, cells [80,124]. Single cell transplantation studies showed that BM or FL HSCs fail to generate tissue-resident B-1a cells, a subset of innate-like lymphocytes which reside primarily in body cavities and mucosa, can secrete IgM, and are able to elicit a fast T-cell independent antibody response [125,126]. Moreover, analysis in Ncx1-null embryos devoid of circulation which lack pre-HSCs in the YS [55] confirmed the presence of T cell potential (including αβ T cells, γδ T cells, and Vγ3 T cells) in the E9.5 YS as well as in the embryo proper [81]. Later fate mapping studies confirmed these findings in vivo [127]. Similar to other progenitors of the second YS wave, both B and T precursors seem to arise from HE. These studies demonstrate heterogeneity in the lymphoid compartment which originates from both HSCs and HSC-independent precursors, the relative contributions of which are difficult to evaluate due to lack of experimental approaches able to differentiate HSC- and HSC-independent lymphoid progeny. In fact, most Cre lines used to label HSCs (i.e., Flt3-Cre, S100a4 or Mds1^CreERT2^) may also label YS-derived lymphoid progenitors [73,128]. The precise identity of HSC-independent precursors harboring lymphoid potential is therefore still unclear. Lympho-myeloid immune-restricted progenitors (LMPs) have been identified as Lin^−^ c-Kit^+^ Flt3^+^ IL7Ra^+^ Rag1^+^ cells initially emerging from HE in the E9.5 YS [79]. Fate mapping studies of Rag1-expressing cells demonstrated a significant contribution of these cells to fetal myelopoiesis (though decreasing from E11.5 to E14.5), as well as a lack of Mk or erythroid contribution, findings also supported by transcriptional analysis [79]. LMPs, and not HSCs, were found to colonize the embryonic thymus independent of Notch signaling [129]; however, the origin of these early thymus-seeding progenitors was questioned by a later report [130]. The true extent of LMP contribution to fetal lymphopoiesis, as well as the lineage relationship of these multi-potent precursors with HSC-independent B- and T-committed progenitors, remain to be determined (Figure 2).

## 4. The Third and Definitive Wave: Not Just HSCs

### 4.1. Heterogeneity within the Pool of Intra-Aortic Clusters

**Figure 3:**
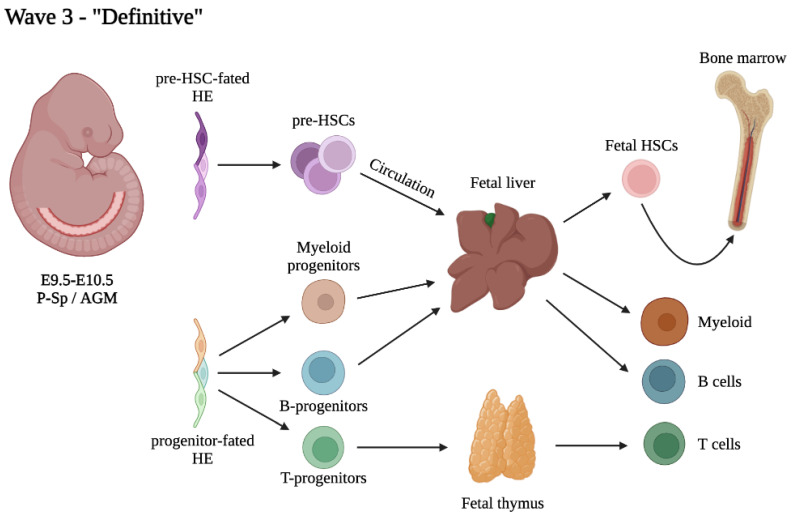
Suggested schematic of the intraembryonic or “definitive” hematopoietic wave.

The onset of the definitive hematopoietic wave (Figure 3) is often associated with the beginning of intra-embryonic hematopoiesis, which takes place around midgestation. This process has been recognized initially by microscopic observation of discrete clusters of hematopoietic cells within the aorta and major arteries [10]. At the same time, the unique ability of the embryonic AGM region to autonomously generate HSCs has been recognized since more than 25 years [12]. HSCs are first generated through EHT from HE localized in the ventral wall of the dorsal aorta, vitelline and umbilical arteries [14,131]. Mature transplantable HSCs can be detected at low frequency in the AGM as soon as E10.5 [8], however, the presence of HSC precursors, the so-called pro/pre-HSCs, has been observed more consistently [15] and can be traced back as early at E9.5 [16]. Rigorous quantification by limiting dilution assays showed that pre-HSC numbers largely exceed HSCs in the AGM and peak at E11, shortly before dropping at E12.5, coincidentally with the appearance of HSCs in the FL [83]. It has now been confirmed that pre-HSCs are localized in intra-vascular hematopoietic clusters [18,132]. However, extrapolation from data available from literature strongly suggests that not all such clusters represent pre-HSCs. Hematopoietic clusters peak in number at E10.5 with 700 to 800 c-Kit^+^ cells in the whole aorta as determined by imaging of whole mouse embryos [17,70]; but only 20 to 30 pre-HSCs are found at the same stage [83]. This means that intra-aortic clusters are heterogeneous in nature, and the majority represent progenitors other than pre-HSCs. In the mouse, hematopoietic clusters are found in both dorsal and ventral part of the aorta and despite a high degree of similarity at the transcriptional level [59], pre-HSCs are found more consistently in ventral clusters [133]. Pro- and Pre-HSCs are known to express the endothelial marker VE-Cadherin, and upregulate CD43 and CD45 along with their maturation [15,16]. It has been shown that markers such as CD201, Hlf, CD27 and Selp can enrich for pre-HSCs [39,57,134,135,136], however to our knowledge, no marker or combination of markers can be used to prospectively identify all pre-HSCs with near 100% accuracy. This could also mean that the pre-HSC subset is intrinsically heterogeneous. Recently, with the aim of defining the transcriptomic signature of the first functional HSCs in the AGM, an iterative single-cell analysis has been performed [39]. Interestingly, confocal microscopy localized HSCs preferentially within small intra-aortic clusters of 1-2 cells [39]. This leaves with the question of what are the remaining cells in intra-aortic clusters, which, as mentioned above, represent their majority. Beyond (pre-)HSCs, are there also HSC-independent progenitors generated as part of the intra-embryonic hematopoietic wave? Indeed, single cell transcriptomics identified two “sub-waves” within intra-aortic clusters, namely one early wave of progenitors with lympho-myeloid potential, followed by pre-HSCs [58]. Consistent with this, half of the intra-aortic clusters are likely to be progenitors with myeloid potential, as determined by CFU-C assays [137]. What is the precise contribution and role of intra-embryonically derived HSC-independent progenitors? To answer this question, we would need to trace the contribution of these progenitors separately from extra-embryonic hematopoietic cells and from pre-HSCs, and for this purpose more specific markers and tools would be needed. Hence, dissecting the heterogeneity of the definitive wave at the moment is technically challenging, but will eventually be required in order to fully unravel the complexity of the ontogeny of the hematopoietic system.

### 4.2. Heterogeneity of Embryonic and Fetal HSCs

HSCs have been defined as a population of cells that share the same key functional property: to be able to fully reconstitute the hematopoietic system upon transplantation for an extended period of time. While initially described as a homogenous population, this is turning out to be an oversimplification of the actual situation. In the last 20 years, more and more levels of heterogeneity are being highlighted. Indeed, the adult HSC pool is heterogeneous as exemplified by different repopulation pattern kinetics seen in single-cell transplantation settings [138,139], and also confirmed as lineage-biased outputs in unperturbed systems (reviewed in [140]). The improvement of surface marker combinations, and in particular the introduction of SLAM markers, has helped dissect the heterogeneity in the HSC and Multi-Potent Progenitor (MPP) compartment, allowing different subpopulations with high purity [141]. Further aspects of diversity in individual HSCs have been found as variations in the ability to self-renew, cell cycle activity, metabolic and transcriptional profile [142]. The determinants of HSC heterogeneity can be both cell-extrinsic (localization to distinct niches) and cell-intrinsic (somatic mutations, epigenetic alterations, variations due to asymmetric division or stochastic processes) [143]. As the heterogeneity of the adult HSC compartment is a wide topic which has been the subject of recent excellent reviews [142,143,144,145], from here we will focus specifically on recent insights concerning the embryonic and fetal HSC compartment.

An elegant demonstration of the fact that functional heterogeneity is already present in the embryonic HSC pool came from a study from the Rodewald lab, which employed a Cre-loxP based barcoding technique (termed “Polylox”) to introduce labels in single AGM HSCs and thus follow their fate in an unperturbed system [146]. This study found that the adult HSC compartment is a composed by a high number of clones of various sizes, in which each of the embryonic HSCs gave rise to hundreds of adult HSCs. The lineage output was varied and largely multi- or oligo-lineage rather than uni-lineage [146]. Further improvements of in vivo barcoding approaches by the same group and from the Camargo lab implemented types of barcodes that are also transcribed, and thus allowing recording of lineage histories and simultaneous transcriptomic profiling of single-labeled cells in unperturbed systems [147,148]. Both studies employed labeling of E9.5 hematopoietic progenitors as a proof of principle. Remarkably, both studies detected clones found only in the HSC compartment, meaning that there is a subset of HSCs which remain inactive under steady state, without generating any detectable progeny (though notably mast cells and innate lymphocytes were not assessed in either study, and Mk were not assessed in the Rodewald study) [147,148]. These “inactive HSCs” expressed markers of LT-HSC quiescence in the adult BM, and appear to be specified early in development. The bulk of adult hematopoiesis is instead established by myeloid biased and multilineage HSCs. Importantly, the transcriptomic landscape of HSCs associated with their in vivo fate. Interestingly, the study from the Camargo group analyzed BM in four different bones and found great variation of clonal outputs between these bones, meaning that fetal HSC expansion potential might be dependent on the specific niche to which they home [147]. Future interrogations using these powerful tools will no doubt offer further insight into drivers of heterogeneity in hematopoiesis.

Despite the difficulty of probing functional and molecular heterogeneity within the AGM HSC compartment due the extreme scarcity of cells (about 1 to 2 functional HSCs/AGM at E11.5 in the mouse [149]), some studies provided relevant insight. BMP and Hedgehog signaling pathways are two of the main molecular determinants of embryonic HSC development [69]. At E11, all AGM HSCs are BMP-activated based on a transgenic marker of BMP activation, when read out directly in vivo (BRE-GFP) [150]. However, explant cultures revealed the presence of HSCs in both fractions [151]. The non-BMP-activated HSCs were further shown to be indirectly dependent on Hedgehog signaling through VEGF [151]. In the E14 FL, most HSCs are BMP-activated but some non-BMP-activated are detected, whereas adult BM HSCs were largely negative for the BMP activation marker [150]. The two cell types appear to be lineage related as GFP^+^ cells can give rise to GFP-, but not vice versa. These two cell states might represent different points in a maturation trajectory. Consistent with this, based on transcriptomic data, BMP4 activated HSPCs are more similar to the hemogenic endothelium, whereas non-BMP4 activated HSPCs are purely hematopoietic. In the BM, the BMP activation state correlates with lineage output [150].

Fetal HSCs are known to bear different characteristics with respect to their adult counterparts [152]. Notably, HSCs in the FL display a higher degree of self-renewal despite being more proliferative [153]. There is evidence of heterogeneity in the FL HSC compartment, which can be linked to ontogeny. Using a Flk2-Cre::Rosa26-mTmG mouse model, a group identified developmentally restricted HSCs which exhibit a strong lymphoid bias [154]. In this model, HSCs which have expressed Flk2 (also known as CD135 or FLT-3) throughout their history will express GFP, whereas HSCs arising a Flk2-independent manner will express tdTomato. The authors found GFP^+^ and tdTomato^+^ cells in E12.5–E14.5 FL HSCs, but all adult HSCs were tdTomato^+^, meaning they never expressed Flk2 in their lineage history. However, when transplanted, FL GFP^+^ HSCs could engraft primary and secondary recipients, with a strong B- and T-cell bias. Further lineage tracing identified a contribution of these fetal-specific HSCs to innate-like B and T lymphoid subsets [154]. Could these developmentally restricted HSCs link to the lymphoid-biased progenitors identified within the AGM cluster cells discussed in the previous paragraph? Further work will be needed to clarify this.

The shift from a “fetal-like” to adult phenotype was found to complete postnatally, at around 3 weeks of age in the mouse [155]. This transition appears to already begin at late fetal stages and its start is accompanied by a spike in type I interferon target gene activation [156]. From there, the switch takes place in a gradual though highly asynchronous way, as shown by multimodal single cell analysis [156]. Hence, for all the stages ranging to the late fetal to the juvenile and early adult age the HSC pool is highly heterogeneous and comprises a mix of HSCs with fetal and adult identities. It will be of great interest to establish how this heterogeneity can predispose to certain age-related diseases.

## 5. Concluding Remarks

The embryonic development of the hematopoietic system is characterized by multiple levels of heterogeneity. Subsequent overlapping waves of hematopoietic precursors with diverse characteristics emerge from different intra-and extra-embryonic locations, in a highly stereotyped fashion. After their initial generation, these progenitors undergo rapid changes, resulting in differentiation, migration and/or proliferation. This makes the developing hematopoietic system highly heterogeneous not only because of the various cell types involved, which can be unique to fetal life, but also because these cells can transition between intermediate cell states, while still retaining functionality. One example is the acquisition of adult characteristics in the HSC compartment, which was shown to function as a gradual but uncoordinated process, which does not complete until adult life [156].

In the present review, we have outlined how the field is tackling the issue of heterogeneity in the development of the hematopoietic system, and has transformed the initial “primitive” and “definitive” view into the current more complex picture. This was largely carried out thanks to new advances in single cell analyses [157], but it is certainly possible that there will be further radical changes in our view of hematopoietic development once new higher resolution technology is developed. One important conclusion that we can draw from the last decade of research is that HSC-independent progenitors have gained a surprising amount of relevance. It is now established that significant parts of the adult hematopoietic compartment are derived from embryonic progenitors other than HSCs. In particular, it is now surfacing that certain tissue resident immune cell populations, whether of the myeloid of lymphoid lineage, primarily retain an embryonic HSC-independent origin, reinforcing the view of the adult immune system as “layered”. These findings, together with the observation that these tissue-resident immune cells can independently self-renew locally, in part challenged the view that HSCs reside at the top of the adult hematopoietic system. Moreover, it is now emerging that ontogeny is a primary determinant of specific and vital functions carried out by these tissue-resident immune populations of embryonic origin. For instance, EMP-derived Mac play a critical role in the establishment of mucosal immunity in the intestine, a function that cannot be performed by BM-derived Mac [158]. Maternal exposure to microbial infections and inflammation can shape the fetal immune system, and therefore have the potential to generate a long-lasting impact by altering the composition of the adult immune compartment [159].

The heterogeneity within the developing hematopoietic system has important implications for the understanding of pediatric leukemia. Ontogeny can determine susceptibility or resistance to transformation in response to the acquisition of a specific genetic lesion [160,161]. Further, HSC-independent progenitors can be considered as possible cells-of-origin in pediatric leukemias [162]. Many childhood B-ALLs express aberrant myeloid markers and undergo lineage switching after treatment, therefore they could arise from a progenitor with both lymphoid and myeloid potential [163]. Supporting this hypothesis, in an hIPS model the presence of the childhood ALL-associated translocation ETV6-RUNX1 blocked the cells in a IL7R^+^ LMP-like progenitor cell state. This impeded the myeloid-to-lymphoid transition seen during ontogeny, and resembled a pre-leukemic phenotype, possibly more susceptible to second hits [164].

There is still a lot to be undertaken in order to reach a complete understanding of hematopoietic development and of the significance of its inherent heterogeneity: for instance, we still don’t know what exactly its main drivers are, and it is likely that we can’t yet appreciate the full extent of its implications. Future studies and technological advances will no doubt help shedding more light on this fascinating topic. In turn, additional insight in developmental hematopoiesis will be instrumental in order to implement ex vivo production of HSPCs for therapeutic purposes, as well as to gain a better understanding of leukemia and immunity.

## Figures and Tables

**Table 1:** Characteristics of EMP-competent and HSC or lymphoid- competent hemogenic endothelium. A green tick icon indicates dependence on a pathway or expression of a marker. A red cross indicates lack of dependence on a pathway or absence of expression of a marker.

	HSC- and Lymphoid- Competent HE	EMP-Competent HE	References
*Notch* pathway dependence	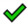	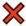	[45,46,47]
Arterial identity dependence	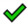 (Lymphoid potential)	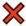	[48,49,50]
WNT canonical pathway dependence	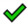	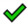	[31]
*Cxcr4* expression	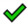 (Lymphoid potential)	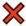	[40]
*Lyve1* expression	Low	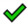	[42,43,44]
*Hlf* expression	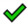	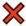 (until E10.5)	[57]
Blood flow dependence	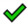	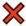	[51,52,53,54,55,56]
Hypoxia/glycolysis	Decrease of hematopoietic output	Increase of hematopoietic output	[55]

## Data Availability

No new data was generated over the course of this study.
